# Evaluation of medication regimen complexity as a predictor for mortality

**DOI:** 10.1038/s41598-023-37908-1

**Published:** 2023-07-04

**Authors:** Andrea Sikora, John W. Devlin, Mengyun Yu, Tianyi Zhang, Xianyan Chen, Susan E. Smith, Brian Murray, Mitchell S. Buckley, Sandra Rowe, David J. Murphy

**Affiliations:** 1 Department of Clinical and Administrative Pharmacy, University of Georgia College of Pharmacy, 1120 15th Street, HM-118, Augusta, GA 30912 USA; 2 Bouve College of Health Sciences, Northeastern University, Boston, MA USA; 3 Division of Pulmonary and Critical Care Medicine, Brigham and Women’s Hospital, Boston, MA USA; 4 Department of Statistics, University of Georgia Franklin College of Arts and Sciences, Athens, GA USA; 5 Department of Pharmacy, University of North Carolina Medical Center, Chapel Hill, NC USA; 6 LaJolla Pharmaceuticals, Waltham, USA; 7 Oregon Health and Science University, Portland, OR USA; 8 Division of Pulmonary, Allergy, Critical Care and Sleep Medicine, Emory University School of Medicine, Atlanta, GA USA

**Keywords:** Health care, Therapeutics

## Abstract

While medication regimen complexity, as measured by a novel medication regimen complexity-intensive care unit (MRC-ICU) score, correlates with baseline severity of illness and mortality, whether the MRC-ICU improves hospital mortality prediction is not known. After characterizing the association between MRC-ICU, severity of illness and hospital mortality we sought to evaluate the incremental benefit of adding MRC-ICU to illness severity-based hospital mortality prediction models. This was a single-center, observational cohort study of adult intensive care units (ICUs). A random sample of 991 adults admitted ≥ 24 h to the ICU from 10/2015 to 10/2020 were included. The logistic regression models for the primary outcome of mortality were assessed via area under the receiver operating characteristic (AUROC). Medication regimen complexity was evaluated daily using the MRC-ICU. This previously validated index is a weighted summation of medications prescribed in the first 24 h of ICU stay [e.g., a patient prescribed insulin (1 point) and vancomycin (3 points) has a MRC-ICU = 4 points]. Baseline demographic features (e.g., age, sex, ICU type) were collected and severity of illness (based on worst values within the first 24 h of ICU admission) was characterized using both the Acute Physiology and Chronic Health Evaluation (APACHE II) and the Sequential Organ Failure Assessment (SOFA) score. Univariate analysis of 991 patients revealed every one-point increase in the average 24-h MRC-ICU score was associated with a 5% increase in hospital mortality [Odds Ratio (OR) 1.05, 95% confidence interval 1.02–1.08, *p* = 0.002]. The model including MRC-ICU, APACHE II and SOFA had a AUROC for mortality of 0.81 whereas the model including only APACHE-II and SOFA had a AUROC for mortality of 0.76. Medication regimen complexity is associated with increased hospital mortality. A prediction model including medication regimen complexity only modestly improves hospital mortality prediction.

## Introduction

Robust mortality prediction models in the intensive care unit (ICU) facilitate clinical decision making, clinical investigation, and quality improvement^1,2^. Severity of illness scores (Acute Physiology and Chronic Health Evaluation [APACHE II], Sequential Organ Failure Assessment [SOFA]) have remained the standard for mortality prediction but have potential limitations^3,4^. Medications are frequently prescribed in the ICU to improve patient outcomes, but critically ill adults are also at high risk for experiencing adverse drug events, some of which are associated with an increased risk for mortality^5^.

The medication regimen complexity-intensive care unit (MRC-ICU) score has been proposed to succinctly characterize medication regimens in the ICU^6,7^. In small, preliminary studies the MRC-ICU demonstrated correlation to illness severity scores (APACHE II and SOFA), hospital mortality, ICU complications (e.g., fluid overload, drug-drug interactions), and the number and intensity of medication interventions performed by critical care pharmacists^6–13^. In a subsequent study of 3,908 ICU adults at 28 centers, every one-point increase in the MRC-ICU score was associated with a 7% increase in the odds of mortality and a 0.25 day increase in ICU length of stay^14^; however, this study did not incorporate adjustments for severity of illness.

Delineating how holistic medication use in the ICU relates to patient mortality in the context of illness severity is an important next step in optimizing pharmaceutical care during critical illness^6^. After characterizing the association between MRC-ICU, severity of illness and hospital mortality, we sought to evaluate the incremental benefit of adding MRC-ICU to illness severity-based ICU mortality prediction models. We hypothesized that incorporation of medication complexity into illness severity-based ICU mortality prediction models would improve hospital mortality prediction.

## Methods

### Study population

This retrospective, observational study was reviewed by the University of Georgia (UGA) Institutional Review Board (IRB) and deemed to be exempt from IRB oversight (Project00001541). All methods were performed in accordance with the ethical standards of the of the UGA IRB and the Helsinki Declaration of 1975. Patient data were obtained via the Carolina Data Warehouse, which houses Epic® electronic health record (EHR) data from the University of North Carolina Health System (UNCHS), an integrated healthcare delivery system. Given the intensity of the data collection effort, particularly for MRC-ICU calculations, we employed random number generation to identify a sample of 1000 adults aged ≥ 18 years admitted to the ICU for ≥ 24 h between October 2015 and October 2020. Only data from the first ICU admission for each patient was included. Patients were excluded if they were placed on comfort care within the first 24 h of their ICU stay.

### Variables

The primary outcome was hospital mortality. The EHR was queried to obtain baseline patient characteristics, medication information, and patient outcomes. Baseline patient characteristics including age, sex, race, admitting diagnosis and ICU type were collected. The admission APACHE-II and SOFA scores (based on values from the first 24 h of ICU admission) were calculated when values for all domains were available^15,16^.

Medication information including drug, dose, route, duration, and timing of administration were recorded. The MRC-ICU score was calculated at 24 h and on each ICU day for up to one week. The MRC-ICU consists of 35 discrete (i.e., medication) items where each is assigned a weighted value and then summed to create a MRC-ICU score for a patient’s medication regimen at any given time point^9^. For example, a patient receiving vancomycin (3 points), a norepinephrine infusion (1 point), and topical chlorhexidine (1 point) on ICU day 2 would have an ICU day 2 total MRC-ICU score of 5. In addition to the mean daily MRC-ICU score, the frequency of each item contributing to the score was calculated.

### Analysis

Descriptive statistics were computed for the relevant variables and a plot of the MRC-ICU score by ICU day and mortality was made to visualize the relationship between mortality, ICU day and MRC-ICU score. Additionally, a component analysis of MRC-ICU was performed to identify the frequency of medication use in the cohort and how it related to hospital mortality.

Multiple logistic regression models with the inclusion of different combinations of MRC-ICU, SOFA, APACHE II and their interactions were developed to evaluate the relationship of MRC-ICU and severity of illness scores on ICU day 1 and hospital mortality. The Variance Inflation Factor (VIF) was calculated during this model-building process to avoid potential concerns with multicollinearity and therefore ensure model reliability and robustness. Odds ratios (OR) were reported alongside their corresponding 95% confidence intervals (CI) for the predictors of interest.

To further evaluate the models, the cohort (containing 521 patients with complete APACHE-II, SOFA, and MRC-ICU data) was split into training and test sets, using a ratio of 4:1. To evaluate the predictive abilities of each model on hospital mortality, area under the receiver operating characteristic curve (AUROC) was computed in addition to sensitivity, specificity, negative predictive value (NPV), and positive predictive value (PPV) in the test set. Results were subsequently compared between AUROCs using DeLong’s test where prediction thresholds were chosen by maximizing Informedness, Matthew’s Correlation Coefficient (MCC) and F1 scores in the training set. A two-sided *p*-value less than 0.05 was used to determine statistical significance for all variables. All analyses were performed using *R* (version 4.1.2).

### Ethical approval

This was an observational study that was reviewed by the University of Georgia Institutional Review Board (IRB) and determined to be exempt from IRB oversight (Project00001541).

## Results

**Figure 1:**
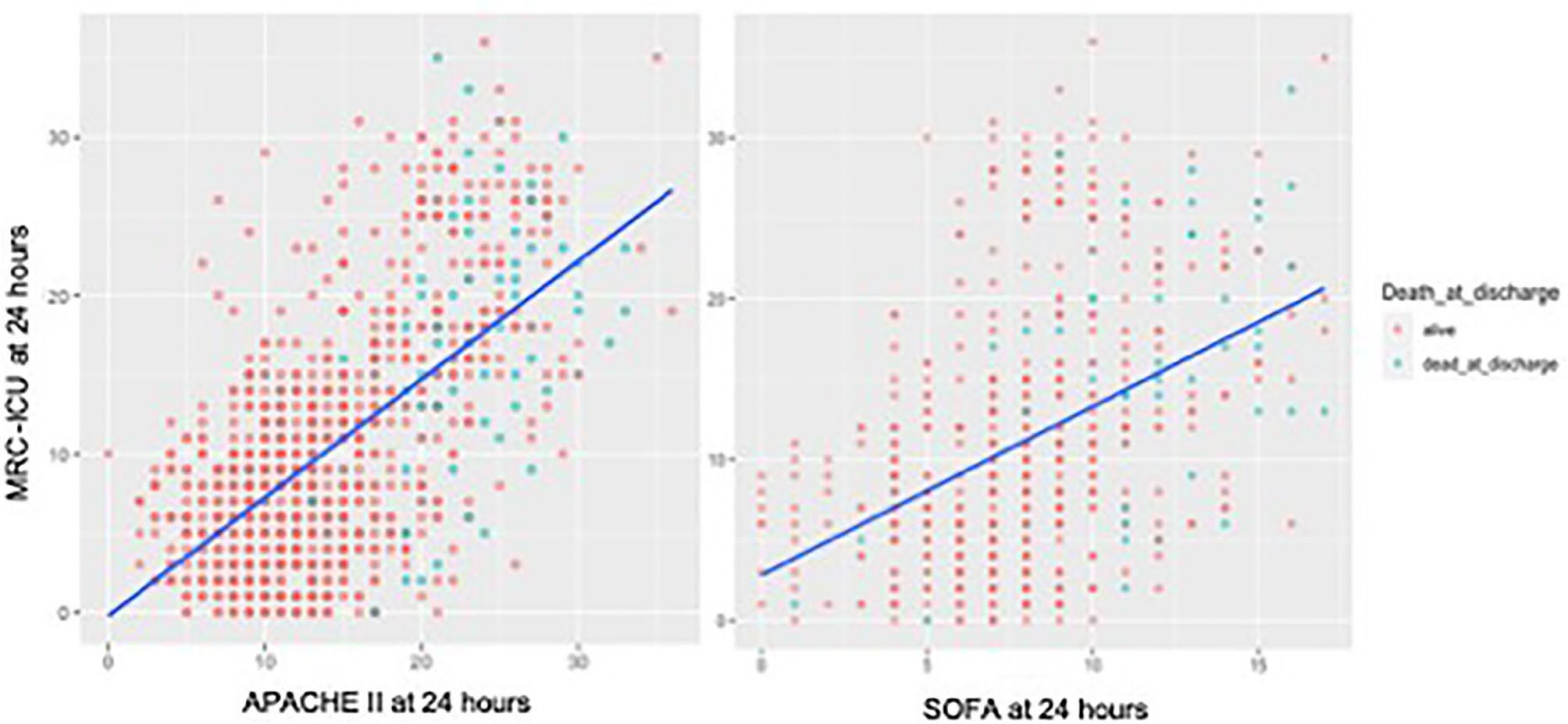
Hospital mortality in relation to MRC-ICU and severity of illness. In the left panel, the blue line indicates the fitted regression line of MRC-ICU versus APACHE II score (i.e., the typical MRC-ICU score of a patient with a certain level of APACHE II score). In the right panel, the blue line indicates the fitted regression line of MRC-ICU versus SOFA score. Colors are set to be 50% transparent, indicating that darker colors have more overlap of patients. All deaths occurred in patients with APACHE II scores over 10, and a possibility exists that those patients with lower MRC-ICU scores had a higher mortality than expected given their APACHE II score.

Of the 38,729 patients admitted to UNCHS during the evaluation period, 7515 (19.4%) met all study criteria. A total of 1000 (13.3%) of these 7515 patients were randomly selected for the study cohort. After excluding an additional 9 patients because the ICU admission did not represent their index ICU admission, a total of 991 patients were included in the final cohort. These patients were 61.2 (standard deviation [SD] 17.5) years old and predominantly medical (40.7%), cardiac (30.8%), surgical (9.8%) and neurological (9.4%). The mean 24-h APACHE II score (based on complete data from 963 patients) was 14.2 (6.3) and the mean 24-h SOFA score (based on complete data from 533 patients) was 8 (6.4). The mean MRC-ICU score at 24 h (based on the 991 patients) was 10.3 (7.7). The overall hospital mortality rate was 9.8%. Table 1 provides a full summary of the study cohort characteristics, and Fig. 1 provides a plot of mortality in relation to MRC-ICU and severity of illness.

**Table 1:** Study population characteristics.

Feature	N = 991
Age	61.2 (17.5)
Female	428 (43.2)
Race	
Caucasian	645 (65.1)
Black	235 (23.7)
Other	111 (11.2)
ICU type	
Medical	404 (40.7)
Cardiac	305 (30.8)
Surgical	97 (9.8)
Neurosciences	93 (9.4)
Burn	70 (7.1)
Other	22 (2.2)
Admission diagnosis	
Cardiovascular	244 (24.6)
Neurology	139 (14.0)
Pulmonary	124 (12.5)
Trauma	111 (11.2)
Infection including sepsis	97 (9.8)
Gastrointestinal	83 (8.4)
Neoplasm	62 (6.3)
Dermatology	59 (6.0)
Renal	47 (4.7)
Endocrine	24 (2.4)
Other	
Use of mechanical ventilation	312 (31.5)
Use of vasopressors	287 (29.0)
Severity scores	
APACHE II at 24 h, mean (SD), n (%)	14.2 (6.3), 963 (97.2%)
SOFA at 24 h, mean (SD), n (%)	8 (6.4), 533 (53.8%)
Patient outcomes	
Mortality	97 (9.8)
ICU length of stay, days	5.1 (9.5)

Data are presented as n (%) or mean (standard deviation (SD)) unless otherwise stated.

*AKI* acute kidney injury; *CRRT* continuous renal replacement therapy; *SOFA* sequential organ failure assessment, *APACHE II* Acute Physiology and Chronic Health Evaluation; *ICU* intensive care unit.

The frequency of each MRC-ICU score component is presented in Supplemental Digital Content (SDC) Supplemental Table 1. The association between each MRC-ICU component and hospital mortality is presented in SDC Supplemental Table 2. The change in MRC-ICU over each ICU day is presented in SDC Supplemental Figure 1, with the highest score observed on ICU day 1. Hospital mortality by MRC-ICU score (as well as baseline APACHE-II and SOFA score) is presented in SDC Supplemental Figure 2. The results of the multiple logistic regression models with different combinations of MRC-ICU, APACHE-II, SOFA score and their interactions for hospital mortality, are presented in SDC Supplemental Table 3 and SDC Supplemental Figure 3.

While the positive relationship between APACHE II or SOFA score and hospital mortality did not change after controlling for MRC-ICU, the relationship between MRC-ICU and hospital mortality changed after controlling for APACHE II or SOFA (Table 2). While the univariate analysis revealed a one-point increase in the MRC-ICU was associated with a 5% increase in the odds of hospital mortality, the multivariate analysis (that accounted for APACHE II and SOFA) found a 6% reduction in hospital mortality for each one-point MRC-ICU increase (OR 0.94, 95% CI 0.90–0.98, *p* = 0.007).

**Table 2:** Univariate and multivariable analysis of MRC-ICU association with mortality.

Mortality	Univariate			Multiple Variable		
	OR	95% CI	*p*-value	OR	95% CI	*p*-value
MRC-ICU at 24 h	1.05	1.02, 1.08	0.002	0.94	0.90, 0.98	0.007
SOFA at 24 h	1.32	1.21, 1.44	< 0.001	1.16	1.04, 1.29	0.006
APACHE II at 24 h	1.17	1.12, 1.23	< 0.001	1.19	1.12, 1.28	< 0.001

The multiple variable model presented includes MRC-ICU, APACHE II, and SOFA scores at 24 h.

*SOFA* sequential organ failure assessment, *APACHE II* Acute Physiology and Chronic Health Evaluation, *ICU* intensive care unit.

*p*-values are obtained from Wald tests for each variable using normal approximation.

**Figure 2:**
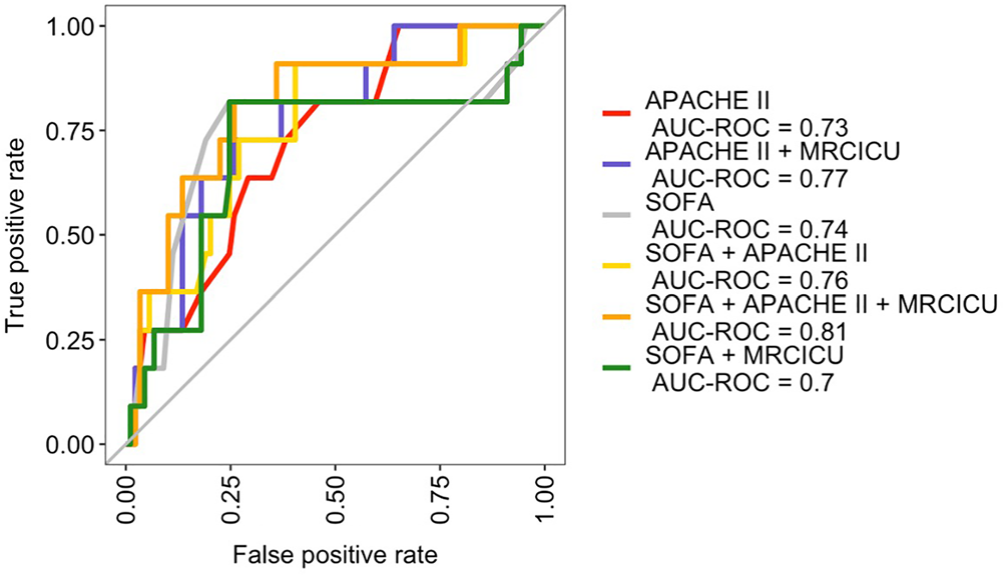
AUROCs for hospital mortality prediction.

The VIF scores for APACHE-II, SOFA, and MRC-ICU were found to be below 5 (2.1, 1.4 and 1.9 respectively) indicating low correlation between these variables and thus an absence of significant multicollinearity within the models. Comparative performance of the APACHE-II, SOFA, and MRC-ICU ICU mortality models is presented in Table 3. AUROC curves were plotted (Fig. 2) for the six models comprising combinations of severity of illness scores and medication data. The addition of medication variables resulted in an AUROC change from 0.76 to 0.81. Negative predictive value exceeded 90% for all mortality models while positive predictive value remained low. Sensitivity, specificity, positive predictive value, and negative predictive values are reported in SDC Supplemental Table 4.

**Table 3:** Comparison of predictive accuracy for logistic regression models of hospital mortality.

Model	Univariate			Multiple variable
	APACHE II	SOFA	MRC-ICU	Model
AUROC	0.73	0.74	0.47	0.81
Brier Score	0.096	0.090	0.10	0.09
Hosmer–Lemeshow chi square	8.54*	10.51	10.57	0.66*

*AUROC* area under the receiver operating characteristic curve, *SOFA* sequential organ failure assessment, *APACHE II* Acute Physiology and Chronic Health Evaluation, *ICU* intensive care unit.

The multiple variable model includes MRC-ICU, APACHE II, and SOFA scores at 24 h.

*Number of bins for Hosmer–Lemeshow chi square was 5; APACHE II and the multiple variable model had models with *p* > 0.05 indicating goodness of fit.

The results of the DeLong testing (SDC Supplemental Table 5) for the training and test sets revealed MRC-ICU inclusion improved both the APACHE-II (*p* = 0.014) and combined APACHE-II and SOFA (*p* = 0.007) mortality prediction models. The results for the threshold analyses (SDC Supplemental Table 6) revealed the APACHE-II, SOFA, and MRC-ICU ICU mortality model to be the most robust across various threshold selection strategies and that it strikes the optimal balance between sensitivity and specificity (SDC Supplemental Figure 4). The calibration plot (SDC Supplemental Figure 5) revealed strong model predictiveness when the predicted score was < 0.25. However, the model tends to under-predict hospital mortality when the predicted score was between 0.25 and 0.50 and tends to over-predict mortality when the predicted score was > 0.5.

## Discussion

After establishing MRC-ICU, APACHE II and SOFA each have a direct association with increased hospital mortality, we found that a severity of illness-based prediction model that includes medication regimen complexity only modestly improved hospital mortality prediction, with this combination resulting in an AUROC threshold exceeding 0.8. While the MRC-ICU continued to show association with hospital mortality, the inclusion of severity of illness resulted in a switch from an increased odds for mortality with increased MRC-ICU to a reduced odds for mortality with increased MRC-ICU. These findings represent the first characterization of the relationship between medication regimen complexity to mortality in the context of severity of illness predictors.

There are a number of possible explanations as to why the addition of the MRC-ICU to APACHE II and SOFA-based model only incrementally improved our ability to predict hospital mortality. Both APACHE II and SOFA are good at predicting mortality. Mortality in the ICU results from many factors that evolve over the course of admission; incorporating medication complexity represents only one factor that may change in a critically ill adult’s ICU trajectory. The MRC-ICU does not represent the ‘ground truth’ for appropriate ICU pharmacologic intervention; we do not know if the medication(s) administered were right or wrong for the patient.

Calculating baseline mortality risk is essential for clinical trials and quality improvement evaluation because it facilitates patient enrollment, evaluation of treatment groups, and comparison of cohorts at different time points^1^. Moreover, it allows for meaningful benchmarking among and within institutions looking to improve quality of care^1,2^. Historically, this mortality risk was inferred through the objective quantification of severity of illness using gold standards like APACHE-II and SOFA scores derived from regression models. Although still widely accepted and used given their relative simplicity of calculation, these approaches assume a degree of linearity that may not be present in the patient with a fluctuating degree of critical illness and can suffer from missing data^17,18^. Technological advancements may support incremental improvement of these metrics, particularly through modeling that allows for additional information to be included and that allows for such non-linear relationships. Thus, adding medication data, which certainly influences patient outcomes, has the potential to modestly improve prediction.

While prior MRC-ICU evaluations have shown a direct relationship between medication regimen complexity and increased mortality, these studies did not account for baseline severity of illness^6–13^. After accounting for severity of illness, medication regimen complexity was associated with decreased mortality. In our cohort, death occurred in patients with the higher APACHE II scores, and mortality appeared to sharply increase in patients with an MRC-ICU score over 10. A possibility exists that an “ideal” level of medication complexity to reduce hospital mortality is present for a given severity of illness.

Our results have implications for clinical decision-making, healthcare workload designations, and future prediction-based research. The inter-related nature of a patient’s baseline severity of illness and their treatment requires further exploration: the possibility exists that the addition of medication variables to mortality prediction models may yield particular relevance in patients whose severity of illness is not so extreme but in a middle ground where medication therapy is most likely to play the most impactful role on outcomes. Although medications are treatment for critical illness, they are also independent risk factors for ICU complications that can adversely affect ICU outcomes. Even then, incremental benefits between ‘a medication’ for a disease and ‘the optimal medication’ for that disease are notable. As such, medications warrant further investigation for use as important predictor variables for ICU outcomes such as mortality, length of stay, and duration of mechanical ventilation. Moreover, our results suggest that the non-linear interaction between illness severity and MRC-ICU as it relates to outcomes is an important consideration that warrants further investigation. Notably, MRC-ICU may be unique because it is solely based on medications being ordered thus precluding the potential for missingness. Thus, the possibility exists that including medications may improve outcome prediction models.

Limitations of our study include a sample size that may be too small to capture all medication-related permutations of heterogenous ICU patients. Additionally, the single center design (and resultant lack of validation test set) limits definitive conclusions. Severity of illness was estimated only at baseline; consideration of daily ICU SOFA and MRC-ICU scores in a time-dependent hospital mortality model might lead to different results. Although it was expected that MRC-ICU and severity of illness would correlate given both previous studies and the general construct (i.e., increasingly complex medication regimens are required for sicker patients) and that incremental value of the addition of medication variables to regression models would be observed, these patterns may be better elucidated by methods other than traditional regression, including artificial intelligence methodologies that are better equipped to handle non-monotone and non-linear relationships and may provide superior calibration^2^. As such, important next steps from this first evaluation of how medication regimen complexity relates to mortality include further evaluation of temporal relationships of MRC-ICU and severity of illness, particularly in the context of machine learning and artificial intelligence methodologies.

## Conclusion

In this study, MRC-ICU was shown to have a relationship with mortality as well as severity of illness. Moreover, severity of illness and medication regimen complexity play an important role in mortality prediction. Further analysis of the complex interplay of these variables is warranted.

## Supplementary Information

Supplementary Information.

## Data Availability

Following request and authorship team approval of the appropriateness of the request, datasets can be made available.
