# Supplementary material for: Evaluation of medication regimen complexity as a predictor for mortality
Source: Sci Rep. 2023 Jul 4;13:10784. doi: 10.1038/s41598-023-37908-1 (PMC10319715; doi:10.1038/s41598-023-37908-1)
Supplement: Supplementary file 1 — Supplementary Information. [file 41598_2023_37908_MOESM1_ESM.docx]

**Supplementary Material**

**Supplemental Figure 1.** Change in the MRC-ICU score by each ICU day.

**Supplemental Figure 2.** Hospital mortality by MRC-ICU, APACHE-II and SOFA score.

**Supplemental Figure 3.** Assessment of the interaction between medication regimen complexity and severity of illness for the training and testing sets.

**Supplemental Figure 4**. Metrics versus threshold plot.

**Supplemental Figure 5.** Calibration plot.

**Supplemental Table 1.** Line-item representation of the MRC-ICU score at 24 hours.

**Supplemental Table 2.** Univariate component analysis of the MRC-ICU score at 24 hours and risk for hospital mortality.

**Supplemental Table 3.** Evaluation of multiple logistic regression for hospital mortality with interaction terms between MRC-ICU, APACHE-II and SOFA scores.

**Supplemental Table 4.** Sensitivity, Specificity, Negative Predictive Value, and Positive Predictive Value for hospital mortality prediction models.

**Supplemental Table 5.** Results of DeLong’s Test for comparisons among AUROC curves for training and test sets.

**Supplemental Table 6.** Results of Negative Predictive Value (NPV) and Positive Predictive Value (PPV) threshold analyses.

**Supplemental Figure 1.** Change in the MRC-ICU score by each ICU day

This figure represents the changes in mean MRC-ICU score (bars indicate standard deviation) over the first 7 days of the ICU stay.

**Supplemental Figure 2.** Hospital mortality by MRC-ICU, APACHE-II and SOFA score**
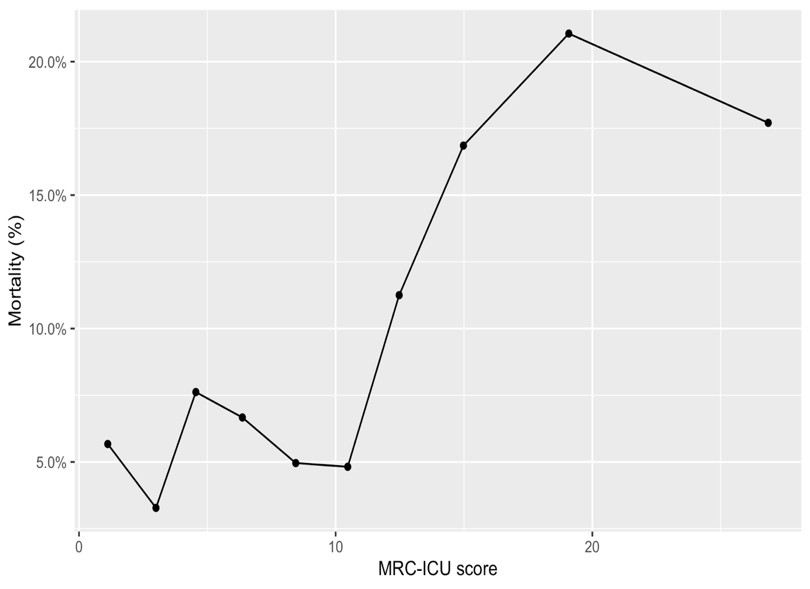
**
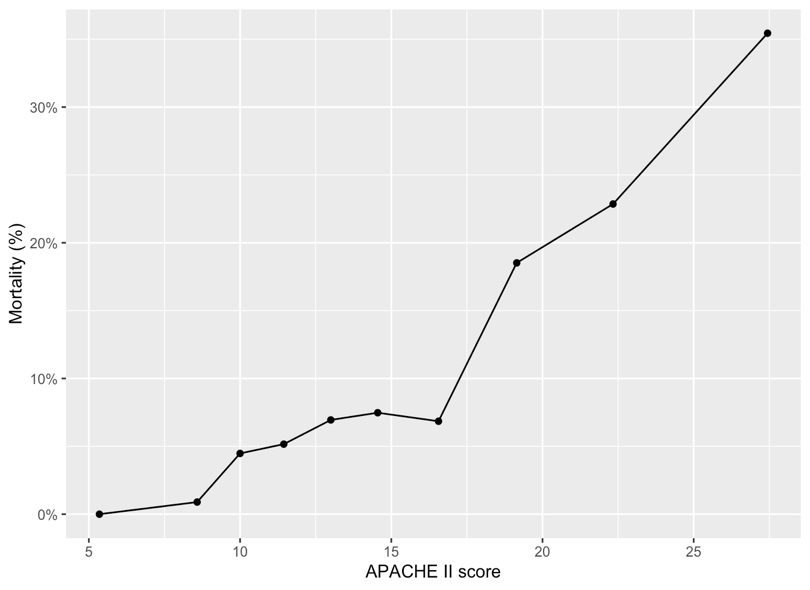

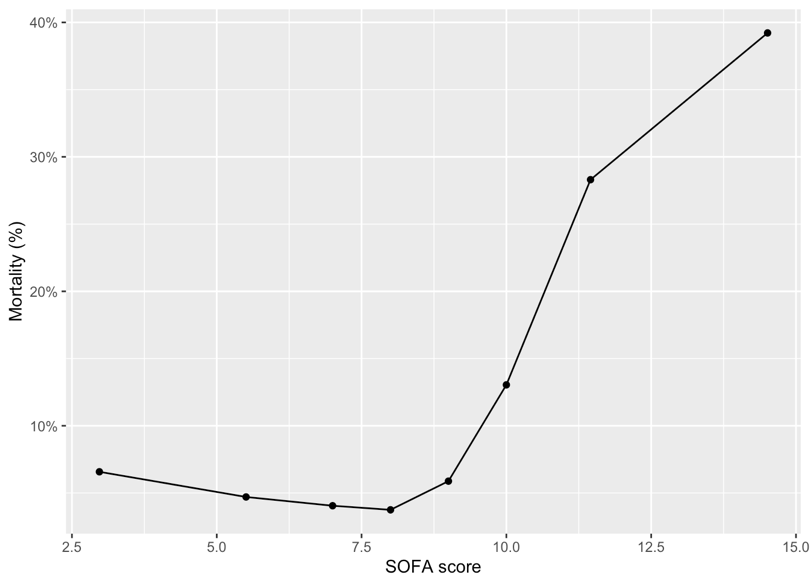


**Supplemental Figure 3.** Assessment of interaction between medication regimen complexity and severity of illness for the training and testing sets

**Supplemental Figure 4.** Metrics versus threshold plot

**Supplemental Figure 5.** Calibration plot

**Supplemental Table 1.** Line-item representation of the MRC-ICU Score at 24 hours

| **Component** | **N = 991**  **n (%)** |  |
| --- | --- | --- |
| **Medications** | |  |
| Aminoglycosides (amikacin, gentamicin, tobramycin) | 4 (0.4) |  |
| Amphotericin B and liposomal amphotericin B | 1 (0.1) |  |
| Antiarrhythmics (amiodarone, dofetilide, sotalol) | 100 (10.0) |  |
| Anticonvulsants (carbamazepine, phenobarbital, phenytoin, valproic acid) | 32 (3.2) |  |
| Antimicrobials | 372 (37.5) |  |
| Azole antifungals (posaconazole, voriconazole) | 10 (1.0) |  |
| Blood products (factor products, antithrombin III) | 11 (1.1) |  |
| Bowel regimen | 420 (42.3) |  |
| Broad Spectrum antimicrobials | 237 (23.9) |  |
| Chemotherapy (active inpatient) | 11 (1.1) |  |
| Chlorhexidine | 241 (24.3) |  |
| Clozapine | 0 (0) |  |
| Continuous infusions (exclude those listed elsewhere) | 631 (63.6) |  |
| Continuous infusion opioids and sedatives | 481 (48.5) |  |
| Digoxin | 17 (1.7) |  |
| Ganciclovir/valganciclovir | 4 (0.4) |  |
| Glycemic control (subcutaneous insulin; exclude IV insulin) | 430 (43.3) |  |
| Hyperosmolar fluids (hypertonic saline (1.5%, 3%, 23.4%), mannitol) | 21 (2.1) |  |
| Immunosuppressants (cyclosporine, sirolimus, tacrolimus) | 20 (2.0) |  |
| Lidocaine (continuous infusion) | 24 (2.4) |  |
| Lithium | 2 (0.2) |  |
| Neuromuscular blockers | 64 (6.4) |  |
| Opioids and sedatives (scheduled and PRN) | 679 (68.5) |  |
| Prostacyclins (epoprostenol, iloprost, treprostinil) | 2 (0.2) |  |
| Stress ulcer prophylaxis | 467 (47.1) |  |
| Theophylline | 1 (0.1) |  |
| Therapeutic heparins (enoxaparin, heparin infusion) | 381 (38.4) |  |
| Thromboembolic prophylaxis | 196 (19.7) |  |
| Total parenteral nutrition | 6 (0.6) |  |
| Vancomycin (IV) | 235 (23.7) |  |
| Warfarin | 14 (1.4) |  |
| **Devices influencing medication dosing** | |  |
| Dialysis | 11 (1.1) |  |
| Extracorporeal membrane oxygenation (ECMO) | 2 (0.2) |  |
| Intra-aortic balloon pump (IABP) / Left ventricular assist device (LVAD) | 15 (1.5) |  |
| Mechanical ventilation | 291 (29.3) |  |
| *Data are presented as n (%) unless otherwise noted* | | |

**Supplemental Table 2.** Univariate component analysis of MRC-ICU score at 24 hours and risk for hospital mortality.

| **MRC-ICU Item** | **Component at 24 hours, n (%)**  **N = 991** | **Hazard Ratio for hospital mortality (95% CI))** | **P-value** |
| --- | --- | --- | --- |
| Aminoglycosides | 4 (0.4) | -- | -- |
| Amphotericin B & Liposomal Amphotericin B | 1 (0.1) | -- | -- |
| Antiarrhythmics | 100 (10.1) | 0.81 (0.37, 1.74) | 0.6 |
| Anticoagulants (oral, fondaparinux) | 34 (3.4) | 1.36 (0.50, 3.65) | 0.5 |
| Anticonvulsants | 32 (3.2) | 1.58 (0.96, 2.59) | 0.07 |
| Argatroban | 1 (0.1) | -- | -- |
| Azole antifungal | 10 (1.0) | 1.35 (0.33, 5.52) | 0.70 |
| Blood products | 11 (1.1) | 2.15 (0.79, 5.87) | 0.13 |
| Chemotherapy | 11 (1.1) | 0.62 (0.12, 3.22) | 0.6 |
| Clozapine | 0 (0) | -- | -- |
| Digoxin | 17 (1.7) | 0.52 (0.07, 3.74) | 0.5 |
| Ganciclovir/valganciclovir | 4 (0.4) | -- | -- |
| Hyperosmolar fluids | 21 (2.1) | 1.85 (1.12, 3.06) | 0.02 |
| Immunosuppressants | 20 (2.0) | 1.31 (0.63, 2.73) | 0.5 |
| Lidocaine | 24 (2.4) | 0.51 (0.07, 3.68) | 0.5 |
| Lithium | 2 (0.2) | -- | -- |
| Prostacyclins | 2 (0.2) | -- | -- |
| Theophylline | 1 (0.1) | -- | -- |
| Therapeutic heparin | 381 (38.5) | 1.14 (0.81, 1.60) | 0.4 |
| Vancomycin | 235 (23.7) | 2.05 (1.37, 3.07) | <0.001 |
| Warfarin | 14 (1.4) | -- | -- |
| Neuromuscular blockers | 64 (6.4) | 1.51 (0.84, 2.72) | 0.2 |
| Continuous infusions | 631 (63.7) | 1.06 (0.98, 1.15) | 0.20 |
| Total parenteral nutrition | 6 (0.6) | -- | -- |
| Thromboembolic prophylaxis | 196 (19.8) | 0.92 (0.56, 1.50) | 0.7 |
| Stress ulcer prophylaxis | 467 (47.1) | 1.48 (0.98, 2.23) | 0.06 |
| Glycemic control | 430 (43.4) | 1.57 (1.05, 2.35) | 0.03 |
| Bowel regimen | 420 (42.4) | 1.11 (0.74, 1.66) | 0.6 |
| Chlorhexidine | 241 (24.3) | 1.52 (1.02, 2.30) | 0.04 |
| Opioids and sedatives | 679 (68.5) | 0.87 (0.76,1.01) | 0.07 |
| Continuous infusion opioids and sedatives | 481 (48.5) | 1.25 (1.03,1.52) | 0.03 |
| Antimicrobials | 372 (37.5) | 1.15 (0.96, 1.38) | 0.13 |
| Broad spectrum antimicrobials | 237 (23.9) | 1.94 (1.47, 2.57) | <0.001 |
| Dialysis | 11 (1.1) | 1.51 (0.48, 4.81) | 0.5 |
| Extracorporeal membrane oxygenation | 2 (0.2) | -- | -- |
| Mechanical circulatory assist devices | 15 (1.5) | 1.78 (0.56, 5.63) | 0.3 |
| Mechanical ventilation | 291 (29.4) | 2.01 (1.34, 3.02) | <0.001 |
| *Univariate cox regression evaluation line item relationship to mortality; certain line items were not evaluated due to low representation in the dataset* | | | |

**Supplemental Table 3.** Evaluation of multiple logistic regression for hospital mortality with interaction terms between MRC-ICU, APACHE-II and SOFA scores

|  | **Odds Ratio** | **95% Confidence Interval** | **p-value** |
| --- | --- | --- | --- |
| **Univariate Logistic Regression** | | | |
| *Severity of Illness + MRC-ICU* | | | |
| MRC-ICU | 1.05 | 1.02, 1.08 | <0.01 |
| SOFA | 1.32 | 1.21, 1.44 | <0.01 |
| APACHE II | 1.17 | 1.12, 1.23 | <0.01 |
| **Multiple Variable Logistic Regression** | | | |
| *Severity of Illness* | | | |
| SOFA | 1.15 | 1.04,1.27 | <0.01 |
| APACHE II | 1.12 | 1.07,1.18 | <0.01 |
| *Severity of Illness + MRC-ICU* | | | |
| MRC-ICU | 0.93 | 0.89,0.98 | <0.01 |
| SOFA | 1.15 | 1.04,1.28 | <0.01 |
| APACHE II | 1.19 | 1.11,1.27 | <0.01 |
| *SOFA + MRC-ICU* |  |  |  |
| SOFA | 1.30 | 1.19,1.44 | <0.01 |
| MRC-ICU | 1.01 | 0.97,1.04 | 0.67 |
| *APACHE II + MRCICU* |  |  |  |
| APACHE II | 1.24 | 1.17,1.32 | <0.01 |
| MRC-ICU | 0.94 | 0.90,0.98 | <0.01 |
| *Severity of Illness + MRC-ICU + MRC-ICU*SOFA Interaction* | | | |
| MRC-ICU | 0.84 | 0.74,0.95 | <0.01 |
| SOFA | 1.20 | 1.12,1.29 | <0.01 |
| APACHE II | 1.00 | 0.83,1.20 | 0.99 |
| MRC-ICU*SOFA | 1.01 | 1.00,1.02 | 0.05 |
| *Severity of Illness + MRC-ICU + MRC-ICU*APACHEII Interaction* | | | |
| MRC-ICU | 1.05 | 0.91,1.22 | 0.48 |
| SOFA | 1.28 | 1.14,1.45 | <0.01 |
| APACHE II | 1.14 | 1.03,1.27 | 0.01 |
| MRC-ICU*APACHEII | 0.99 | 0.98,1.00 | 0.10 |
| *Severity of Illness + MRC-ICU + MRC-ICU*APACHEII, MRC-ICU*SOFA Interactions* | | | |
| MRC-ICU | 0.98 | 0.84,1.14 | 0.87 |
| SOFA | 1.47 | 1.27,1.71 | <0.01 |
| APACHE II | 0.81 | 0.65,1.01 | 0.06 |
| MRC-ICU*SOFA | 0.98 | 0.97,0.99 | <0.01 |
| MRC-ICU*APACHEII | 1.02 | 1.01,1.03 | <0.01 |
| *Severity of Illness + MRC-ICU + MRC-ICU*APACHEII, MRC-ICU*SOFA, APACHEII*SOFA Interactions* | | | |
| MRC-ICU | 0.96 | 0.79, 1.17 | 0.73 |
| SOFA | 0.86 | 0.59, 1.25 | 0.41 |
| APACHE II | 1.51 | 0.23, 1.88 | <0.01 |
| MRC-ICU*SOFA | 1.03 | 1.01, 1.04 | <0.01 |
| MRC-ICU*APACHEII | 0.98 | 0.97, 0.99 | <0.03 |
| SOFA*APACHEII | 0.99 | 0.98, 1.02 | 0.74 |
| *SOFA: sequential organ failure assessment, APACHE II: Acute Physiology and Chronic Health Evaluation; ICU: intensive care unit* | | | |

**Supplemental Table 4.** Sensitivity, Specificity, Negative Predictive Value, and Positive Predictive Value for hospital mortality prediction models

|  | **AUROC** (95% CI) | **Sensitivity** (95% CI) | **Specificity** (95% CI) | **PPV** (95% CI) | **NPV** (95% CI) |
| --- | --- | --- | --- | --- | --- |
| **APACHE II + SOFA + MRC-ICU** | 0.81, 0.65-0.97 | 0.81, 0.52-0.95 | 0.74, 0.64-0.82 | 0.28, 0.16-0.45 | 0.97, 0.9-0.99 |
| **APACHE II + MRC-ICU** | 0.77, 0.60-0.94 | 0.72, 0.43-0.9 | 0.74, 0.64-0.82 | 0.25, 0.14-0.43 | 0.95, 0.88-0.99 |
| **SOFA + MRC-ICU** | 0.70, 0.52-0.88 | 0.81, 0.52-0.95 | 0.75, 0.65-0.83 | 0.29, 0.16-0.47 | 0.97, 0.9-0.99 |
| **SOFA + APACHE II** | 0.76, 0.59-0.93 | 0.90, 0.62-0.98 | 0.59, 0.49-0.69 | 0.21, 0.12-0.36 | 0.98, 0.9-1 |
| **APACHE II** | 0.73, 0.55-0.91 | 0.81, 0.52-0.95 | 0.53, 0.44-0.64 | 0.18, 0.1-0.31 | 0.96, 0.87-0.99 |
| **SOFA** | 0.74, 0.57-0.91 | 0.81, 0.52-0.95 | 0.75, 0.65-0.83 | 0.29, 0.16-0.47 | 0.97, 0.9-0.99 |
| MRC-ICU: Medication regimen complexity-intensive care unit; AUROC: area under the receiver operating characteristic; CI: confidence interval; PPV: positive predictive value; NPV: negative predictive value | | | | | |

**Supplemental Table 5.** Results of DeLong’s Test for comparisons among AUROC curves for training and test sets

**Training Set**

|  | | | | | | |
| --- | --- | --- | --- | --- | --- | --- |
| **Models compared** | **AUROC of model 1** | **AUROC of model 2** | **z statistic** | **p value** | **adjusted**  **p value** |  |
| APACHE II vs. SOFA | 0.806 | 0.739 | 2.081 | 0.037 | 0.561 |  |
| APACHE II vs. APACHE II + MRCICU | 0.806 | 0.817 | -1.391 | 0.164 | 1 |  |
| APACHE II vs. SOFA + MRCICU | 0.806 | 0.742 | 2.061 | 0.039 | 0.589 |  |
| APACHE II vs. SOFA + APACHE II | 0.806 | 0.801 | 0.342 | 0.732 | 1 |  |
| APACHE II vs. SOFA + APACHE II + MRCICU | 0.806 | 0.817 | -0.904 | 0.366 | 1 |  |
| SOFA vs. APACHE II + MRCICU | 0.739 | 0.817 | -2.255 | 0.024 | 0.362 |  |
| SOFA vs. SOFA + MRCICU | 0.739 | 0.742 | -0.386 | 0.7 | 1 |  |
| SOFA vs. SOFA + APACHE II | 0.739 | 0.801 | -3.085 | 0.002 | 0.031 |  |
| SOFA vs. SOFA + APACHE II + MRCICU | 0.739 | 0.817 | -3.214 | 0.001 | 0.02 |  |
| APACHE II + MRCICU vs. SOFA + MRCICU | 0.817 | 0.742 | 2.163 | 0.031 | 0.459 |  |
| APACHE II + MRCICU vs. SOFA + APACHE II | 0.817 | 0.801 | 0.896 | 0.37 | 1 |  |
| APACHE II + MRCICU vs. SOFA + APACHE II + MRCICU | 0.817 | 0.817 | 0 | 1 | 1 |  |
| SOFA + MRCICU vs. SOFA + APACHE II | 0.742 | 0.801 | -3.114 | 0.002 | 0.028 |  |
| SOFA + MRCICU vs. SOFA + APACHE II + MRCICU | 0.742 | 0.817 | -3.022 | 0.003 | 0.038 |  |
| SOFA + APACHE II vs. SOFA + APACHE II + MRCICU | 0.801 | 0.817 | -1.959 | 0.05 | 0.753 |  |

**Test Set**

| **Models compared** | **AUROC of model 1** | **AUROC of model 2** | **z statistic** | **p value** | **adjusted**  **p value** |
| --- | --- | --- | --- | --- | --- |
| APACHE II vs. SOFA | 0.726 | 0.740 | -0.146 | 0.884 | 1 |
| APACHE II vs. APACHE II + MRCICU | 0.726 | 0.772 | -2.467 | 0.014 | 0.204 |
| APACHE II vs. SOFA + MRCICU | 0.726 | 0.704 | 0.243 | 0.808 | 1 |
| APACHE II vs. SOFA + APACHE II | 0.726 | 0.758 | -0.830 | 0.407 | 1 |
| APACHE II vs. SOFA + APACHE II + MRCICU | 0.726 | 0.809 | -1.934 | 0.053 | 0.796 |
| SOFA vs. APACHE II + MRCICU | 0.740 | 0.772 | -0.331 | 0.741 | 1 |
| SOFA vs. SOFA + MRCICU | 0.740 | 0.704 | 1.802 | 0.072 | 1 |
| SOFA vs. SOFA + APACHE II | 0.740 | 0.758 | -0.281 | 0.778 | 1 |
| SOFA vs. SOFA + APACHE II + MRCICU | 0.740 | 0.809 | -1.023 | 0.306 | 1 |
| APACHE II + MRCICU vs. SOFA + MRCICU | 0.772 | 0.704 | 0.758 | 0.449 | 1 |
| APACHE II + MRCICU vs. SOFA + APACHE II | 0.772 | 0.758 | 0.366 | 0.714 | 1 |
| APACHE II + MRCICU vs. SOFA + APACHE II + MRCICU | 0.772 | 0.809 | -1.037 | 0.300 | 1 |
| SOFA + MRCICU vs. SOFA + APACHE II | 0.704 | 0.758 | -0.982 | 0.326 | 1 |
| SOFA + MRCICU vs. SOFA + APACHE II + MRCICU | 0.704 | 0.809 | -1.679 | 0.093 | 1 |
| SOFA + APACHE II vs. SOFA + APACHE II + MRCICU | 0.758 | 0.809 | -2.711 | 0.007 | 0.101 |

**Supplemental Table 6.** Results of Negative Predictive Value (NPV) and Positive Predictive Value (PPV) threshold analyses

| Model metric scores and 95% CIs with threshold choosing by Informedness | | | | | | |
| --- | --- | --- | --- | --- | --- | --- |
|  | **Threshold** | **Sensitivity** | **Specificity** | **PPV** | **NPV** |  |
| APACHE II | 0.1378 | 0.55, 0.28-0.79 | 0.74, 0.64-0.82 | 0.21, 0.1-0.38 | 0.93, 0.85-0.97 |  |
| APACHE II + MRCICU | 0.1265 | 0.64, 0.35-0.85 | 0.78, 0.68-0.85 | 0.26, 0.13-0.45 | 0.95, 0.87-0.98 |  |
| SOFA | 0.1448 | 0.82, 0.52-0.95 | 0.75, 0.65-0.83 | 0.29, 0.16-0.47 | 0.97, 0.9-0.99 |  |
| SOFA + APACHE II | 0.1304 | 0.64, 0.35-0.85 | 0.74, 0.64-0.82 | 0.23, 0.12-0.41 | 0.94, 0.86-0.98 |  |
| SOFA + APACHE II + MRCICU | 0.1240 | 0.73, 0.43-0.9 | 0.78, 0.68-0.85 | 0.29, 0.15-0.47 | 0.96, 0.88-0.99 |  |
| SOFA + MRCICU | 0.1371 | 0.82, 0.52-0.95 | 0.73, 0.63-0.81 | 0.27, 0.15-0.44 | 0.97, 0.9-0.99 |  |

| Model metric scores and 95% CIs with threshold choosing by Mathews Correlation Coefficient (MCC) and F1 score | | | | | | |
| --- | --- | --- | --- | --- | --- | --- |
|  | **Threshold** | **Sensitivity** | **Specificity** | **PPV** | **NPV** |  |
| APACHE II | 0.1378 | 0.55, 0.28-0.79 | 0.74, 0.64-0.82 | 0.21, 0.1-0.38 | 0.93, 0.85-0.97 |  |
| APACHE II + MRCICU | 0.1321 | 0.64, 0.35-0.85 | 0.79, 0.69-0.86 | 0.27, 0.14-0.46 | 0.95, 0.87-0.98 |  |
| SOFA | 0.1823 | 0.73, 0.43-0.9 | 0.81, 0.72-0.88 | 0.32, 0.17-0.52 | 0.96, 0.89-0.99 |  |
| SOFA + APACHE II | 0.1463 | 0.55, 0.28-0.79 | 0.75, 0.65-0.83 | 0.21, 0.1-0.4 | 0.93, 0.85-0.97 |  |
| SOFA + APACHE II + MRCICU | 0.1240 | 0.73, 0.43-0.9 | 0.78, 0.68-0.85 | 0.29, 0.15-0.47 | 0.96, 0.88-0.99 |  |
| SOFA + MRCICU | 0.2182 | 0.27, 0.1-0.57 | 0.85, 0.77-0.91 | 0.19, 0.07-0.43 | 0.9, 0.82-0.95 |  |
